# Distinct Hepatic Macrophage Populations in Lean and Obese Mice

**DOI:** 10.3389/fendo.2016.00152

**Published:** 2016-12-06

**Authors:** Rafael Mayoral Monibas, Andrew M. F. Johnson, Olivia Osborn, Paqui G. Traves, Sushil K. Mahata

**Affiliations:** ^1^Merck Research Laboratories, Kenilworth, NJ, USA; ^2^CIBERehd – Networked Biomedical Research Center, Hepatic and Digestive Diseases, Madrid, Spain; ^3^Department of Medicine, Division of Endocrinology and Metabolism, University of California San Diego, La Jolla, CA, USA; ^4^Molecular Neurobiology Laboratory, The Salk Institute, La Jolla, CA, USA; ^5^Metabolic Physiology & Ultrastructural Biology Laboratory, Department of Medicine, VA San Diego Healthcare System, San Diego, CA, USA; ^6^Metabolic Physiology & Ultrastructural Biology Laboratory, Department of Medicine, University of California San Diego, La Jolla, CA, USA

**Keywords:** obesity, insulin resistance, inflammation, hepatocytes, Kupffer cells, immunometabolism

## Abstract

Obesity is a complex metabolic disorder associated with the development of non-communicable diseases such as cirrhosis, non-alcoholic fatty liver disease, and type 2 diabetes. In humans and rodents, obesity promotes hepatic steatosis and inflammation, which leads to increased production of pro-inflammatory cytokines and acute-phase proteins. Liver macrophages (resident as well as recruited) play a significant role in hepatic inflammation and insulin resistance (IR). Interestingly, depletion of hepatic macrophages protects against the development of high-fat-induced steatosis, inflammation, and IR. Kupffer cells (KCs), liver-resident macrophages, are the first-line defense against invading pathogens, clear toxic or immunogenic molecules, and help to maintain the liver in a tolerogenic immune environment. During high fat diet feeding and steatosis, there is an increased number of recruited hepatic macrophages (RHMs) in the liver and activation of KCs to a more inflammatory or M1 state. In this review, we will focus on the role of liver macrophages (KCs and RHMs) during obesity.

## Introduction

The rising prevalence of obesity represents a major global health challenge, not least because it is considered a significant risk factor for a wide array of non-communicable diseases. Prominent among these are diseases of the liver, ranging from steatosis through to cirrhosis, collectively termed non-alcoholic fatty liver disease (NAFLD) (1). However, the etiology linking obesity with liver pathology is incompletely understood, hindering attempts to treat these conditions.

A landmark discovery offering therapeutic potential for the metabolic syndrome was the finding that the adipose tissue of obese mice and humans displays hallmarks of an inflammatory state, including increased concentrations of tumor necrosis factor alpha (TNF-α) and increased monocyte/macrophage infiltration (2–4). Indeed, TNF-α is sufficient to induce features of the metabolic syndrome, such as insulin resistance (IR), and many chemical and genetic depletion studies have demonstrated the importance of inflammation and inflammatory macrophages in this process [recently reviewed in Ref. (5)]. Macrophage accumulation also occurs in other key metabolic tissues including muscle (6–9), liver (10–12), and pancreas (13, 14), which contribute to the dysregulation of glucose homeostasis. In this review, we focus on the composition and behavior of hepatic macrophage populations in obese mice and highlight recent advances that could aid in the targeting of this axis to treat aspects of the metabolic syndrome.

## The Liver at the Interface between Metabolism and Immunity

The liver is a key metabolic organ, which regulates a variety of processes vital for maintaining metabolic homeostasis. These include control of glucose production and lipid metabolism, dysregulation of which are symptomatic of the metabolic syndrome. The liver also plays key roles as part of the immune system secreting acute-phase proteins, complement components, cytokines, chemokines, and being positioned, along with the gastrointestinal tract, at the major interface between ourselves and our external, even microbial environment (15, 16). This unique position where metabolism and immunity are intertwined is reflected in the liver architecture, whereby immune cells are intimately connected to hepatocytes and liver sinusoidal endothelial cells (LSECs) (17, 18), as well as the cross-regulation whereby metabolic stress can result in hepatic immune activation leading to metabolic dysregulation (19, 20).

The liver maximizes nutrient absorption as blood flows through a system of sinusoidal vessels and fenestrations through beds of hepatocytes (17). The majority of blood within the sinusoid derives from the intestines *via* the hepatic portal vein and is rich in both nutrients, and also potentially immunogenic microbial molecules, or in cases of opportunistic infection microbes themselves (17). Therefore, in addition to facilitating nutrient absorption, sinusoids must also enable the removal of immunogenic material and allow the immune system to combat of infection. Kupffer cells (KCs) are located in the hepatic sinusoids and play a key role in this process (18). They bind a range of microbes or microbial ligands *via* microbe-associated molecular patterns (MAMPs), and by phagocytosis prevent them penetrating into the general circulation (18). Lipopolysaccharide (LPS), for example, is readily detectable in portal blood, but only rarely detectable in systemic circulation (21). Compared with macrophages from other locations, KCs are predisposed to respond to activation signals in a less inflammatory fashion and are especially characterized by producing high concentrations of the anti-inflammatory cytokine, interleukin 10 (IL-10) (22). Furthermore, KCs, along with other antigen-presenting cells in the liver, express low levels of co-stimulatory molecules required to initiate an adaptive immune response and high levels of molecules that suppress T cell activation, such as programed death-ligand 1 (PDL-1) (17). Thus, during homeostasis KCs in collaboration with other hepatic immune cell populations clear microbial material while maintaining the inflammatory tone of the liver at a level sufficient for essential functions such as pathogen killing, tissue remodeling, and sinusoidal permeability, but below that which would result in overt inflammation and tissue damage (5, 18, 23). The factors maintaining KCs in this tolerogenic state are not completely clear but are critically important when we consider how these cells and the hepatic macrophage pool in general are altered during obesity.

The phenotype of tissue macrophages is thought to be dependent on their respective ontogeny, as well as their respective polarization state in the tissue environment (24). Polarization was most clearly described by *in vitro* studies, which used cytokines to induce different extremes of macrophage phenotype classified as M1 or classically activated macrophages, considered more pro-inflammatory, and M2 or alternatively activated macrophages that have an anti-inflammatory tone (25). M1 macrophage differentiation can be induced by interferon gamma (IFN-γ), alone or with microbial products such as LPS or inflammatory cytokine TNF-α. In contrast, interleukin 4 (IL-4), interleukin 10 (IL-10), interleukin 13 (IL-13), interleukin 33 (IL-33), transforming growth factor beta (TGF-β), and granulocyte colony-stimulating factor (G-CSF) activate macrophages to differentiate to M2. However, given the range of factors now known to influence macrophage polarization, including cellular metabolic state (26), it is likely that a spectrum of macrophage phenotypes occur *in vivo* even within the same tissue macrophage pool (25). In lean mice, KCs have an M2-like phenotype maintained by the type 2 cytokine, IL-4, and the nuclear hormone receptor peroxisome proliferator activator receptor delta (PPAR-δ) (27, 28). Thus, KCs are specialized by virtue of their derivation from the yolk sac early in development (24, 29), and by factors in the liver environment, which maintain them in a less inflammatory, M2-like state (27, 28).

## Parenchymal and Non-Parenchymal Cells in Liver

**Figure 1:**
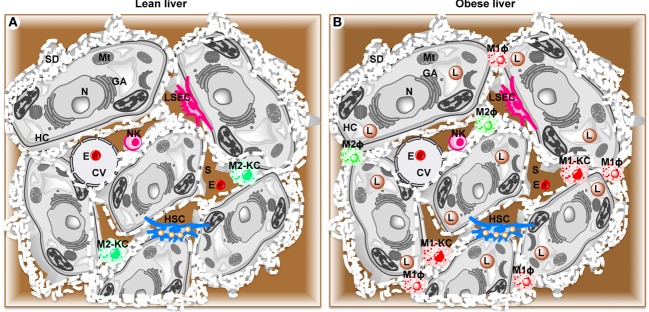
**Schematic diagram showing parenchymal and non-parenchymal cells in liver**. **(A)** Lean liver showing parenchymal hepatocytes (HC) and non-parenchymal anti-inflammatory Kupffer cells (M2-KC), natural killer cells (NK), hepatic stellate cells (HSC), and liver sinusoidal endothelial cells (LSEC). **(B)** High fat diet-induced obese liver showing activated pro-inflammatory Kupffer cells (M1-KC), recruited hepatic macrophages (RHM), and lipid droplets (L). CV, central vein; E, erythrocyte; GA, Golgi apparatus; Mt, mitochondria; N, nucleus; S, sinusoid.

Hepatocytes are the major parenchymal cells, while the non-parenchymal cells integrate five cell populations including resident macrophages or KCs (30), recruited hepatic macrophages (RHMs), resident innate lymphocytes or natural killer cells (NKs) (31, 32), fat storing cells termed Ito or stellate cells (HSCs) (33), and LSECs (34) (Figure 1).

These non-parenchymal cell populations can be identified by a variety of cell surface markers. In general, KCs and RHMs both express epidermal growth factor-like module-containing mucin-like hormone receptor-like 1 (F4/80) (35), NKs form two pools distinguished by mutually exclusive expression of CD49a or DX5 (36), HSCs express glial fibrillary acidic protein (GFAP) (37, 38), and LSECs express CD34 (39). In addition, these liver cell populations can also be distinguished by their physical location within the liver and specific ultrastructural characteristics. For example, hepatocytes contain many microvilli, which project into space of Disse (perisinusoidal space) between the endothelial cells and hepatocytes. KCs (~15% of all liver cells) represent the largest population of tissue macrophages (80–90% of resident macrophages in the whole body) (40). KCs are found attached to the luminal surface or inserted in the endothelial lining of hepatic sinusoids (41, 42), which make them the first macrophages to come into contact with gut-derived foreign and potentially noxious material. The size and function of KCs also depend on their specific location in the liver (43) with KCs in periportal regions being larger and more phagocytic with higher lysosomal enzyme activity than KCs in midzonal and perivenous locations (44). Unlike hepatocytes, KCs are amoeboid in shape. Fenestrae form open connections between the lumen of the sinusoid and the space of Disse (45). The transport and exchange of fluid, solutes, and particles between the sinusoidal lumen and the space of Disse containing the parenchymal cell surface are believed to occur through these open fenestrae (46). While KCs utilize phagocytosis to incorporate large particles such as erythrocytes and bacteria, they take up small particles and molecules *via* pinocytic vesicles (47–50). NKs reside in sinusoids and eliminate virus-infected or transformed cells and regulate adaptive immune responses *via* contact-dependent signals and the secretion of cytokines (36, 51–53). HSCs are perisinusoidal cells, which contain characteristic lipid droplets. HSCs maintain vitamin A homeostasis as they store 80% of total vitamin A in the body. Inflammatory signals transform HSCs into myofibroblasts, resulting in collagen production and development of liver fibrosis (54, 55). LSECs possess a high-rate, high-capacity system to remove colloids and water-soluble waster macromolecules from the circulation (34, 56). At the ultrastructural level, LSECs constitute the only mammalian endothelial cells that combine non-diaphragmed fenestrae with a discontinuous basement membrane, which allows blood plasma to enter the space of Disse.

## Liver Macrophage Populations During Obesity

During the course of obesity, the adipose tissue’s ability to store excess energy is compromised, leading to ectopic lipid accumulation in non-adipose tissues such as muscle and liver (57). Intracellular lipid accumulation in ectopic tissues is associated with a phenomenon known as lipotoxicity, which induces cell death, cytokine secretion, and activation of inflammatory processes, especially in the liver (58, 59). Furthermore, dietary stress and obesity can lead to excessive activation of the hepatic immune system due to increased penetration of microbial material (60–62). The response of the liver to damage and inflammation is a complex process involving parenchymal (hepatocytes) and non-parenchymal cells (KCs, NKs, HSCs, and LSECs), as well as monocyte-derived hepatic macrophages, RHMs (12, 63). The failure to regulate this inflammation during the progression of the obesity causes pathological chronic hepatic inflammation characterized by the advance of fatty liver to steatohepatitis, fibrosis, cirrhosis, and eventually liver failure (18, 64). Depletion of phagocytic cells in the liver (including both KCs and RHMs) through the administration of either liposome-encapsulated clodronate or gadolinium chloride protects against high-fat- or high-sucrose-induced steatosis, inflammation, and IR, demonstrating critical role of hepatic macrophages in the development of metabolic dysfunction (65).

## Macrophage Regulation During NAFLD/NASH

**Figure 2:**
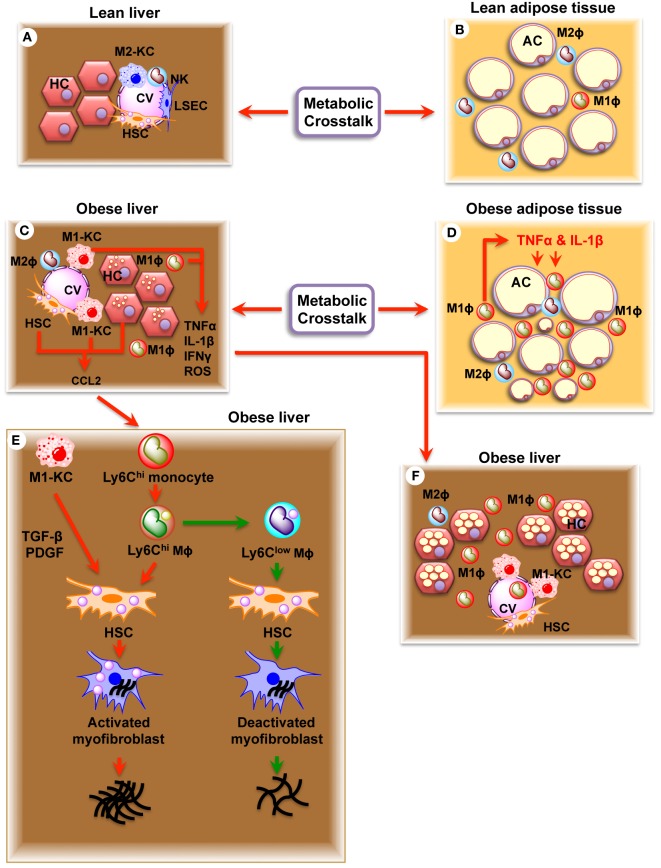
**Schematic diagram showing the effects of resident (KC) and recruited hepatic macrophages (Ly6C^high^) in regulation of non-alcoholic fatty liver disease (NAFLD) and fibrosis**. **(A)** Healthy liver showing parenchymal hepatocytes (HC) and non-parenchymal Kupffer cells (M2-KC), natural killer cells (NK), hepatic stellate cells (HSC), and liver sinusoidal endothelial cells (LSEC). **(B)** Healthy adipose tissue showing adipocytes (AC), adipocyte macrophage 1 (ATM1), and ATM2 macrophages. **(C)** Obese liver showing accumulation of lipid droplets in hepatocytes (HC), activated Kupffer cells (M1-KC), and activated hepatic stellate cells (HSC). Note increased production of TNF-α, IL-1β, IFNγ, ROS, and CCL2. **(D)** Obese adipose tissue showing larger adipocytes (AC), infiltrated ATM1 macrophages, and increased production of TNF-α and IL-1β. **(E)** Obese liver showing NAFLD and NASH. **(F)** Obese liver showing fibrosis. Increased production of CCL2 recruits Ly6C^high^ monocytes, which convert to Ly6C^high^ macrophages inside the liver. Ly6C^high^ macrophages produce TGFβ, connective tissue growth factor (CTGF), and PDGF, which act on HSC and transform HSC to activated myofibroblast. Activated myofibroblast in turn results in fibrosis. Ly6C^high^ macrophage is transformed into Ly6C^low^ macrophage upon eating dead hepatocytes and erythrocytes. Ly6C^low^ macrophage deactivates activated myofibroblasts and decrease fibrosis.

Hepatic lipid accumulation and peroxidation leads to chronic hepatocyte endoplasmic reticulum (ER) stress, the production of reactive oxygen species (ROS), and toll-like receptor (TLR) activation, which converts KCs into an M1 phenotype defined by production of pro-inflammatory cytokines, oncostatin, and prostaglandins (Figure 2) (20, 66, 67). Circulating cytokines, adipokines, and free fatty acids (FFAs) released from inflamed adipose tissue in the obese state or immunogenic material derived from an altered intestinal microbiota can also contribute to KC polarization. M1-KCs secrete chemokine (C-C motif) ligand 2 (CCL2), pro-inflammatory cytokines (TNF-α, IL-1β, and IL-6), macrophage inflammatory protein (MIP)-1a, MIP1b, RANTES, oncostatin, and prostaglandins (PGE_2_), which contribute to the alteration of the liver homeostasis and worsen the hepatic inflammatory response (29). PGE_2_ regulates cytokine production (IL-1, IL-6, TNF-α, and TGF-β) (68, 69), acts synergistically with IL-6 to induce IR (70), and induces production of oncostatin M (OSM) in KCs (71). Increased OSM contributes to hepatic IR and the development of non-alcoholic steato hepatitis (NASH) (71). High levels of TNF-α released by M1-KCs stimulate hepatic expression of CCL2 (also known as MCP1), a powerful monocyte chemoattractant, which recruits CCR2^+^Ly6C^high^ monocytes from the vasculature into the liver (72), where they differentiate into Ly6C^high^ macrophages. The Ly6C^high^ macrophages amplify the severity of obesity-induced inflammation and hepatic IR through the secretion of TNF-α and interleukin 6 (IL-6) (12). C-C chemokine receptor type 2 (CCR2)-deficient mice are protected against weight gain and display reduced development of obesity, illustrating the importance of this chemokine system (73). Once established, this vicious circle of immune cell attraction, infiltration and activation, hepatocyte injury, and further inflammation promotes and defines the pathophysiology of NASH (74).

## Macrophage Regulation of Hepatic Fibrosis

Fibrosis is increasingly appreciated as a major contributor to metabolic dysregulation in obese humans and type 2 diabetic patients (75). Both KCs and recruited Ly6C^high^ macrophages contribute to the development of hepatic fibrosis. KCs activate HSCs through increased production of pro-fibrotic cytokine TGF-β and platelet-derived growth factor (PDGF) (76) leading to fibrosis. Ly6C^high^ macrophages also interact with HSCs to promote fibrosis through increased production of TGF-β, connective tissue growth factor (CTGF), and PDGF (77). Therefore, inhibition of monocyte recruitment through depletion of the pro-inflammatory signal CCL2 results in attenuation of liver fibrosis (77–79). In addition, pharmacological inhibition of CCL2 by the RNA-aptamer mNOX-E36 attenuates liver fibrosis, thereby strengthening a pro-fibrotic function of Ly6C^high^ macrophages (80, 81).

## Macrophage Surface Markers

Due to the distinct functions of RHMs and KCs in suppressing or perpetuating the immune activation (29, 82), it is important to be able to clearly isolate pure populations of each cell type. However, distinguishing RHM from KC has proven difficult mainly due to technical difficulties in isolating and identifying macrophages from the obese liver. KCs (CXCR1^−^) appear histologically as larger cells with multiple phagocytic granules and have been defined by surface marker expression as CD45^+^/CD11c^−^/F4/80^high^/CD11b^low^ (12, 83). RHMs (CXCR1^+^) are smaller than KC, contain fine granules in the cytoplasm, and have been defined by surface marker expression as F4/80^dim^/CD45^+^/CD11b^+^/CCR2^+^ (10), CD11b^+^/Ly6C^high^/Ly6G^−^ (83), or CD45^+^/CD11c^−^/F4/80^low^/CD11b^high^ markers (83) depending on the publication. However, these factors alone do not sufficiently identify pure KC or RHM populations as there is significant size and surface marker overlap with other cell populations, including dendritic cells (DCs), eosinophils, and undifferentiated monocytes (84). KCs, unlike RHMs, have the unique ability to survive to lethal irradiation (85), which has enabled studies into these distinct cell types. The result of these investigations suggests that the number of KCs remains unchanged during the course of obesity, whereas accumulation of RHMs increases several-fold (12). Transcriptome analysis of these RHM and KC populations isolated from lean and diet-induced obese (DIO) mice revealed statistically marked differences between the two cell types on both diets. Furthermore, the Gene Ontology analysis of these transcriptomes showed a restricted list of 16 KC marker genes and 11 RHM markers genes differentially expressed from lean to DIO mice that could provide the opportunity for direct isolation strategies using specific surface markers (12). Interestingly, factors secreted in the culture media from isolated high fat diet (HFD)-RHMs, but not from isolated HFD-KCs, can promote hepatic glucose output and attenuate insulin’s normal inhibitory effects on this aspect of hepatic metabolism suggesting that RHMs are the dominant immune cell type inducing hepatic IR (12, 82).

## Hepatic Gene Expression Changes During Obesity

To identify potential mechanisms underlying the development of obesity and diabetes, many studies have been conducted to characterize changes in hepatic gene expression (86–91). Complex phenotypes such as obesity and IR involve many different interacting biological pathways, but recent technological advances in high throughput sequencing have greatly improved our ability to quantitatively detect gene expression changes in an unbiased way. Investigation of the hepatic gene expression profiles in obese db/db (leptin receptor deficient) mice compared with control mice revealed significant changes in lipid metabolism, gluconeogenesis, mitochondrial dysfunction, and oxidative stress (88, 89). Similar studies using HFD feeding to generate obesity resulted in increased hepatic expression of genes involved in fatty acid catabolism and ketone body synthesis, such as acyl-CoA oxidase1 (*Acox1*) and HMG-CoA lyase (*Hmgcl*), while genes involved in lipogenesis and cholesterol synthesis, such as fatty acid synthase (*Fasn*) and acetyl-CoA synthetase 2 (*Acsl6*), were drastically decreased in the HFD group (86). Further studies also identified upregulation of hepatic gluconeogenic genes and downregulation of expression of lipogenic genes in diabetic Zucker rats (92), with activation of distinct transcriptional regulatory networks during diabetic progression (93).

Due to the practical limitations in obtaining human liver tissue, the most detailed hepatic expression studies have, so far, been conducted in rodent models (86–89, 92, 93). However, with the increasing use of gastric bypass surgery in obese patients, obtaining liver biopsies has become more feasible (91). Comparison of hepatic gene expression before and after weight loss in morbidly obese women identified differentially expressed genes involved in lipid and energy homeostasis, pro-inflammatory tissue repair, and bile acid transport (91). Liver samples from morbidly obese patients with all stages of NAFLD and controls were analyzed by array, and NAFLD specific expression differences were seen for nine genes involved in intermediate metabolism including pyruvate carboxylase (*Pc*), ATP citrate lyase (*Acly*), and phospholipase C-gamma-1 (*Plcg1*) as well as insulin/insulin-like signaling including insulin-like growth factor-1 (*Igf1*), insulin-like growth factor binding protein 2 (*Igfbp2*), and protein kinase C epsilon (*Prkce*) (94). In additional studies, comparison of transcriptional profiles from NASH patients versus non-obese controls also revealed significant changes in genes involved in metabolism, insulin signaling, and inflammation (90). For example, high levels of the central enzyme controlling unesterified arachidonic acid levels of Acyl-CoA synthetase long chain family member 4 (*Acsl4*) and lower levels of insulin signaling genes including *Igfbp2* were observed in NASH versus non-obese controls (95).

Therefore, many hepatic gene expression studies in rodents and humans have been conducted at the level of the whole liver, but whether these changes occur within the hepatocyte or non-parenchymal cells is yet to be fully investigated. Increased understanding of the changes induced in the obese state in the hepatocytes, liver-resident macrophages, and each immune cell population may allow us to specifically target potentially harmful populations while promoting anti-inflammatory populations (96). These studies will also help clarify the molecular mechanisms behind the development of IR and identify potential targets for therapeutic intervention. Furthermore, future integration of transcriptomics data with metabolomics and proteomics data will further our understanding of the mechanisms behind obesity-associated liver disease and help identify biomarkers for the development of disease progression (89).

## Conclusion and Future Perspectives

Although KCs are reemerging in obesity and metabolic syndrome as a critical player in the onset of hepatic IR, as well as NAFLD, their role in metabolism is still largely unknown. We are yet to define the direct role of KCs in metabolic diseases as well as their interactions with neighboring cells and distant organs that modulate liver function and whole body metabolism. After a hepatic insult, KCs secrete important factors involved in the recruitment and transformation of blood monocytes, which are involved in the subsequent development of the hepatic IR. During obesity, the inflammatory state in the liver is associated with a large increase in RHMs with a M1 phenotype, targeting specifically these immune cells or manipulating the activation of KC may be an effective therapeutic strategy in obesity-related chronic liver and NASH. The use of new technologies such as next-generation or single-cell sequencing at different stages of obesity and IR and approaches to isolate and identify the diverse macrophage population and profile their transcriptomes in the liver could provide the opportunity for a direct targeting strategy using specific surface markers. Further research in the field of immunometabolism, including a better understanding of how changes in the microbiota affect the development of inflammation and more knowledge about the factors that direct the polarization state of macrophages toward either the pro- or anti-inflammatory state, is necessary to design new therapeutic strategies for treating T2D and NAFLD.

## Author Contributions

SM and RM conceived the idea. RM, AJ, OO, PT, and SM contributed equally to researching the data and writing of the manuscript.

## Conflict of Interest Statement

The authors declare that the research was conducted in the absence of any commercial or financial relationships that could be construed as a potential conflict of interest.
